# Knowledge, Attitude and Practice of Emergency Contraception Among Graduating Female Students of Jimma University, Southwest Ethiopia

**Published:** 2010-07

**Authors:** Nasir Tajure, B Pharm

**Affiliations:** Department of Pharmacology and Therapeutics, Jimma University, Tel: +2519-11-68-05-76 P.O.BOX 378 , Email: zenastaj@yahoo.com

**Keywords:** Emergency contraceptive, knowledge, attitude, practice, Jimma University

## Abstract

**Background:**

Emergency contraception refers to methods that women can use to prevent pregnancy after unprotected sexual intercourse, method failure or incorrect use. Unwanted pregnancy followed by unsafe abortion can be avoided by using different contraceptive methods including emergency contraceptives. The objective of this study was to assess the knowledge, attitude and practice of emergency contraception among graduating female students of Jimma University main campus.

**Methods:**

A cross-sectional study was conducted in Jimma University main campus in 2009. The calculated sample size was allocated to each faculty proportions to size of female students. Then within the faculty the sample unit was selected by using simple random sampling technique. Data was collected using self administered questionnaire and analyzed using SPSS for widow version 16.0.

**Results:**

A total of 389 (96.5%) volunteered graduating female students participated in the study. One hundred sixty three (41.9%) were ever heard of Emergency Contraceptive, only 11(6.8%) used the method. The common sources of information were friends 60 (36.5%), radio 37 (22.8%) and television 20 (12.3%). One hundred sixteen (71.2%) agreed to use Emergency Contraceptive when they practice unintended sexual intercourse.

**Conclusion:**

Awareness and use of emergency contraception among graduating female students of Jimma University was low. There is a need to educate adolescents about emergency contraceptives, with emphasis on available methods and correct timing of use.

## Introduction

Emergency contraception (EC) is any method of contraception which is used after intercourse and before the potential time of implantation (1). Since it is difficult to determine the infertile time of the cycle with certainty, EC better be provided to any woman who is concerned about her risk of pregnancy regardless of the cycle day of exposure (2).

In 1995, worldwide, woman experienced over 300 million unwanted pregnancies resulting in over 700,000 of them to death because of pregnancy related cases. World Health Organization (WHO) estimated that 84 million unwanted pregnancies occur annually world wide. A worldwide study conducted by the WHO to assess the reproductive needs of the population found unexpected discrepancy between the young peoples familiarity with modern contraception and on the other hand the high levels of unwanted pregnancy and unsafe abortion experienced. Millions of women who could benefit from emergency contraception have never hearted of it (3, 4).

Unintended pregnancy poses a major challenge to the reproductive health of young adults in developed countries (5). About 3 million unwanted pregnancies occur in the United States. Most of these results are from nonuse of contraception or from noticeable contraceptive failure, (such as broken condom) which could be prevented with the use of EC (6).

In several African countries, survey among University students showed that only three quarter of youth had heard about EC, and minimal accurate knowledge about its use (7, 8).

Unsafe abortion is a major medical and public health problem in Ethiopia. Ethiopia has a high incidence of unwanted pregnancies and unsafe abortions, particularly among adolescents (9). Teen age girls are particularly prone to unintended pregnancies as studies revealed that a relatively high percentage (16.3%) of teenage women were already pregnant or have given birth previously (10). Unsafe abortion is one of the top causes incriminated in the high number of maternal morbidity and mortality in Ethiopia (11).

Thus, understanding of knowledge, attitude and practice of EC is critical for countries like Ethiopia with a population policy aiming at reducing unwanted pregnancy. Unfortunately little research has been conducted in this area in the country. This study will show the scope of the problem in the study area and information gathered from this study will provide baseline data for further study. Furthermore, this study will provide baseline data to assist policy makers in developing appropriate evidence-based strategies to promote the use of emergency contraceptive pills in Ethiopia.

The aim of this study is to examine the knowledge, attitude and practice of graduating female students of Jimma University main campus regarding emergency contraception.

## Subjects and Methods

A cross-sectional study was conducted to assess KAP of 2009 graduating female students of Jimma University main campus. The University trains professionals at undergraduate and post graduate level through innovative community based (CBTP), team based (TTP) and research based (SRP) education approach. There were eight faculties and one college in the university during the study period. The total number of graduating female students from the main campus in 2009 was 498. The study was conducted in January, 2009.

Sample size was determined taking the following assumptions; since there was no previous study in the area, the proportion of students who are aware of emergency contraception to be 50%, confidence interval of 95%, margin of error 5% and 5% non response rate was taken. The sample size, therefore, was 403. Then the sample was allocated to each faculty in the main campus by using simple random sampling method. Accordingly the distribution appeared, Education faculty (91), Medical faculty (51), Technology faculty (17), Public Health faculty (43), Business and Economics faculty (115), Humanity and Social Sciences faculty (38), Natural and Information System faculty (15) and Law faculty (33).

Data were collected using a structured pre-tested self-administered questionnaire which was enclosed in an unmarked envelope. The questionnaires contained information about socio demographic characteristic of the students (age, sex, marital status, educational level, religion, their faculty year, of study at university), knowledge, practice and attitude towards emergency contraceptives.

We determined the knowledge about ECPs using four multiple-choice questions. Each correct question corresponded to 1 point, and so there was a total of 4 points for the four questions. Students were considered to have adequate knowledge if they scored 3 or 4 out of 4. They were considered to have inadequate knowledge if they scored between 0, 1 and 2 out of 4.

The students' attitudes were measured using seven items rated on a three-point Likert scale as (1) agree (A), (2) neutral (N) and (3) disagree (D). Using this three-point scale for seven questions, we arbitrarily set the maximum score for each respondent at 21 and the minimum at seven. We decided that a high score was indicative of positive attitude while a low score would be indicative of a negative attitude. The student's practice were measured whether used ECs or not.

Data were then coded, checked for completeness and consistency. Then the data were entered and analyzed using SPSS for windows version 16.0. For descriptive statistics results were expressed in terms of proportions or percentages and association between variables was calculated using Chi-square test and p-value of <0.05 was considered as significant.

A formal letter written from school of pharmacy, Jimma University to Student Research Program (SRP) and permission was obtained and given to registrar office of the university. Consent was obtained from the respondents and brief explanation of aim of study was provided with the questionnaire. Only those who volunteered were included in the study. Strict confidentiality was assured through anonymous recording and coding of questionnaires and placed in safe place.

## Results

Three hundred eighty nine (389) were included in the study giving a responses rate of 96.5%. Most of the respondents 267 (68.64%) were within age group of 20–24 years. The majority 373 (95.8%) were not currently married. Two hundred fifty four (65.3 %) were orthodox Christians, 258 (66.3 %) originally from rural area and 309 (79.5%) studying 3 year undergraduate program (Table 1)

**Table 1:** Socio-demographic characteristics and faculty distribution of participants, Jimma University, January 2009.

Character sties		Number (n=389)	Percent
Age			
	15–19	71	18.3
	20–24	267	68.7
	25–30	43	11.0
	31–49	8	2.0
Marital status			
	Single	373	95.8
	Married	15	3.9
	Divorced	1	0.3
	Widowed	0	0.0
Area of origin			
	Urban	131	33.7
	Rural	258	66.3
Religion			
	Orthodox	254	65.3
	Muslim	53	13.6
	Protestant	74	19.0
	Catholic	5	1.3
	Other£	3	0.8
Faculty			
	Medical	49	12.7
	Technology	15	4.0
	Public health	41	10.4
	Education	89	22.8
	Business and Economics	114	29.3
	Humanity and social science	36	9.2
	Natural and information system	12	3
	Law	33	8.56
Duration of program			
	Three year	309	79.5
	Four year	67	17.2
	Five year or more	13	3.3

£ Pagan, Waqefeta

Regarding to respondents knowledge about emergency contraception (EC), 163 (41.9%) ever heard or knew EC; their common sources of information were friends for 60 (36.5%), radio for 37 (22.8%) and television for 20 (12.3%) (Table 2).

**Table 2:** Sources of information for ECPs among the graduating female students who know ECs in Jimma University main campus, January 2009.

Source		Number	Percent
	Friends	60	36.5
	Radio	37	22.8
	TV	20	12.3
	News paper	16	10.0
	Health education	19	11.4
	Others	11	7.0
	Total	163	100

Of those respondents who had heard of emergency contraceptives 39 (24%) correctly identified progesterone only pills while 12 (7.4%) identified combined oral contraceptive as an emergency contraceptive method. Forty nine (30%) correctly identified the recommended 72 hours as the time limit for emergency contraceptive pills. Forty four (27.1%) and 43 (26.6%) of the respondents identified the recommended doses and the recommended time between doses, respectively (Table 3).

**Table 3:** Knowlegde regarding the timing and dosing of emergency contraceptive among graduating female students who know ECs in Jimma University, January 2009.

Knowledge questions		Number	Percent
Recommended time to take ECPs (n=163)			
	With in 24 hours after sex	14	8.3
	With in 48 hours after sex	31	19.2
	With in 72 hours after sex	49	30.0
	I don't know	69	42.5
Recommended number of dose (n=163)			
	One dose	34	21.0
	Two dose	44	27.1
	Three dose	7	4.2
	I don't know	78	47.7
Recommended time between the doses (n=163)			
	12 hours apart	43	26.6
	24 hours apart	24	14.7
	I don't know	96	58.7
Recommended time for IUCD on emergency contraception (n=163)			
	With in 24 hours after sex	9	6.0
	With in 72 hours after sex	17	10.2
	With in 5 day after sex	48	29.3
	I don't know	89	54.5

From those 163 respondents who knew about ECs, 80(49.2%) responded that ECs could be available in Pharmacies, 45(27.6%) in the Hospitals and 31(19.02%) in Health Centers. Of those 163 students who knew about ECs, 116(71.2%) agreed to use ECs when they practice unintended sexual intercourse, 103 (63.2%) gave their opinion to advise friends to use ECs, 125(76.7%) of respondents replied to agree with increment of prevalence of HIV/AIDS and other STIs when ECs use in the society increases. Worries with use of ECs included, ECs will promote promiscuity 60(36.8%); ECs will affect ongoing regular methods of contraception negatively 39(25.7%) and fear of side effects in using ECs 48(29.4%) (Table 4).

**Table 4:** Attitude towards emergency contraceptive among the graduating female students of JU, January 2009.

Opinions	Agree No (%)	Neutral No (%)	Disagree No (%)
If I have unintended sexual intercourse, I would use ECPs.	116(71.2)	29(17.8)	18(11.0)
If a close friend or relative have unintended sexual inter course	103(63.2)	29(17.8)	31(19.0)
I would advice her to use ECPs.			
Wide spread use of ECPs will increase the prevalence of HIV/AIDS and other STIs.	125(76.7)	27(16.6)	11(6.7)
Emergency contraception promotes promiscuity	60(36.8)	44(26.8)	59(36.4)
Emergency contraception is one way of abortion	60(36.9)	23(14.1)	80(49.0)
I don't want to use ECPs for fear of side effects	48(29.4)	33(20.2)	82(50.4)
Emergency contraception will affect ongoing regular methods of contraception negatively	39(23.9)	42(25.7)	82(50.4)

Of those respondents who had heard of emergency contraceptives, only 11 (6.8%) used ECs. Only oral contraceptive pills were used as an emergency contraception.

General awareness about ECs was significantly associated with faculty of respondents and origin of residence (P value <0.05) (Table 5).

**Table 5:** General awareness of ECs by some selected variables among graduating female students in JU, January 2009.

Variables		Know ECPs	Not know ECPs	Total	P-value

		No (%) N=163	No (%) N=226	No (%) N=389	
Age					
	15–19	38 (23.2)	72 (31.9)	110 (28.2)	P=0.022
	20–24	108(66.0)	123 (54.4)	231(59.5)	
	25–30	14 (8.7)	26 (11.5)	40(10.3)	
	31–49	3 (2.1)	5 (2.2)	8 (2.0)	
Area of origin					
	Urban	93 (57.1)	38 (16.8)	131 (33.7)	P=0.000
	Rural	70 (42.9)	188 (83.2)	258 (66.3)	
Religion					
	Orthodox	116 (71.3)	138 (61.0)	254 (63.5)	
	Muslim	17 (10.5)	36 (16.0)	53 (13.6)	P= 0.180
	Protestant	28 (17.9)	46 (20.4)	74 (19)	
	Catholics	2 (0.12)	3 (1.3)	5 (1.3)	
	Others	0 (0.0)	3 (1.3)	3 (0.8)	
Faculty					
	Medical	42 (27.7)	7 (3.1)	49 (12.7)	
	Technology	5 (3.1)	10 (4.4)	15 (4.0)	
	Public Health	33 (20.3)	8 (3.5)	41 (0.44)	P=0.000
	Education	28 (17.2)	61 (27.0)	89 (22.8)	
	Business & Economics	31 (19.1)	83 (36.8)	114 (29.3)	
	Humanity & social science	10 (6.7)	26 (11.5)	36 (9.3)	
	Natural & Information	4 (2.4)	8 (3.5)	12 (3.0)	
	Sciences Law	10 (6.1)	23 (10.2)	33 (8.5)	

## Discussion

Although emergency contraception is not recommended as a regular family planning method, it is a useful method after unprotected sexual intercourse to reduce the chance of unwanted pregnancies (1). Emergency contraception is most useful when there is a failure of barrier methods such as slippage and breakage of condoms, or when sexual intercourse was unpremeditated (6).

The result from this study revealed that only two fifth of the respondents had heard of the method. This is lower than the reports on university students in Hong Kong (70%) (11) and Jamaica (84%) (12). This could be due to low health promotion and low availability of EC in developing country like Ethiopia. It is comparable to reports from Uganda (45.1%) (8), South Africa (42%) (7) and Nigeria (48%) (13).

The most common sources of information were friends and radio which is in agreement with report from Uganda among university students in which the main source was friends (34%), health institutions (24.8%) and schools (19.4%) (8). Even from those who had basic awareness of EC, they lacked detailed knowledge about the regimen, how it is taken and its effectiveness in reducing the chances of pregnancy. Only one-third of them have identified the correct timing of administration of pills after unprotected sexual contact which is lower than in the finding from South Africa (42%) (7). The possible reason for the lack of detailed knowledge on this subject may be linked to the source of information; friends /peers that may not have a good grasp of the subject. Our finding is very far reports from countries where there is school sex education program where 95% Finish high school students (14) and 98% among Princeton University students had adequate knowledge of EC (15). The low level of awareness in this study suggests lack of any educational program and service promotion on emergency contraception

Our finding showed that more than 2-thirds of students who knew about EC believed that they would use EC after unprotected sexual intercourse and 63% of them agreed to advice friends or relatives to take emergency contraceptives after unprotected sexual intercourse which is in agreement with reports from Honduras (16). However, a considerable proportion of respondents reported their fear on using EC and misconception. These includes wide spread use of ECs will increase the prevalence of HIV/AIDS and other STIs, emergency contraception promotes promiscuity, and emergence contraception will affect regular methods of contraception negatively. Very few proportion of female students practiced EC in this study like a report from Hong Kong (11). Practice of EC among participants of this study is very low when compared with studies done in community where awareness for emergency contraception is widespread and service is widely available among secondary school students in Scotland (17) and Finland high school students (14). The possible reason for low EC practice rate in this study could be lack of awareness of the place where it is available, lack of correct information, low promotion and availability of the methods in most health institutions.

General awareness about ECs was significantly associated with faculty of respondent and residents area (P<0.05). The respondents from medical and public health faculty and urban area were more likely to have heard of ECs. This could be related to their education background, as respondents from health related faculties could have better chance, and respondents from urban area are more likely to get access to different sources of information for ECPs which gave them higher awareness relatively.

In conclusion, this study showed that the awareness of emergency contraception among the regular graduating female students was low. Even among those who were aware, the detail knowledge and practice of EC was very low. There is a need to educate adolescents about emergency contraceptives, with emphasis on available methods and correct timing of use. There should be promotion of emergency contraceptives to enhance their use and making them easily accessible in hospital, pharmacies and student clinic with. Moreover, health education program should be set up to the university students to avail accurate information about emergency contraception.
